# Noodles, the all-in-one system for on-target efficiency analysis of CRISPR guide RNAs

**DOI:** 10.1016/j.mex.2023.102481

**Published:** 2023-11-15

**Authors:** Dongfa Lin, Syeda Sadia Najam, Yu Liu, Nicola Murgia, Ilya A. Vinnikov

**Affiliations:** a Key Laboratory for Molecular Enzymology and Engineering, School of Life Sciences, Jilin University, Changchun, China; b Laboratory of Molecular Neurobiology, Sheng Yushou Center of Cell Biology and Immunology, Department of Genetics and Developmental Biology, School of Life Sciences and Biotechnology, Shanghai Jiao Tong University, Shanghai, China

**Keywords:** CRISPR-Cas9, Plasmid, On-target activity, Split luciferase, Homology-directed repair, Noodles: CRISPR on-target efficiency analyzer

## Abstract

The efficiency of clustered regularly interspaced short palindromic repeats (CRISPR) guide RNA (gRNA) targeting is critical for CRISPR associated protein 9 (Cas9)-dependent genomic modifications. Here, we developed Noodles, an all-in-one system to test the on-target activity of gRNAs easily and efficiently. Single-strand annealing repair mechanism of the split luciferase gene allows a positive selection of gRNAs efficiently driving nuclease activity of Cas9 from *Streptococcus pyogenes* (SpCas9). Our system can reliably validate *in silico*-predicted gRNAs before implementing them for *in vitro* and *in vivo* applications. Altogether, Noodles might be an asset for researchers and bioengineers, saving their time and efforts, while keeping the screening efficient and sensitive.

• All-in-one dual-luciferase system to easily probe on-target activity of gRNAs based on homology-directed repair mechanism.
• Easy-to-subclone spCas9-based 2-plasmid system comprising Renilla luciferase for transfection efficiency control.

## Graphical abstract

**Figure:**
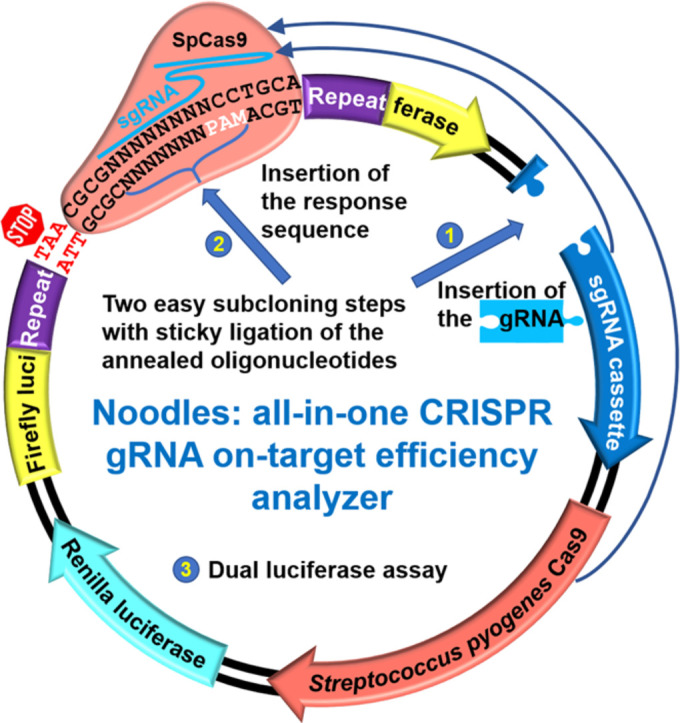

Specifications table

**Table:** 

Subject area:	Biochemistry, Genetics and Molecular Biology
More specific subject area:	Genome engineering
Name of your method:	Noodles: CRISPR on-target efficiency analyzer
Name and reference of original method:	Cradick, T. J., C. J. Antico and G. Bao (2014). “High-throughput cellular screening of engineered nuclease activity using the single-strand annealing assay and luciferase reporter.” Methods Mol Biol 1114: 339–352. [1]
Resource availability:	All resources described in this publication are available on demand. Noodles plasmid is accessible in Addgene.org under #212613

## Background

Using accurate genetic alterations is crucial both for deciphering the roles of genetic elements in (patho)physiology and applications in biotechnology and personalized medicine. CRISPR-Cas9 (clustered regularly interspaced short palindromic repeats-CRISPR associated 9) system has revolutionized genome editing due to its higher versatility, selectivity, efficiency and ease of use compared to the preceding technologies such as zinc-finger nucleases and TALENs [2]. In *Streptococcus pyogenes*, the adaptive immune system molecule SpCas9 binds gRNA via trans-activating CRISPR (tracr) RNA and scans DNA until it matches gRNA leading to the double-stranded cleavage of the target DNA three nucleotides upstream of the NGG sequence [3]. This latter sequence is called protospacer adjacent motif (PAM) and is the key determinant of the binding specificity [4]. Thus, using spacer arrays containing different gRNAs acquired from previous viral infections, bacteria are capable to recognize and neutralize infecting bacteriophages resembling those previous viral strains [5]. Scientists further developed and improved this system, shortening and combining the tracrRNA containing in repeats of these arrays with artificial gRNAs [6]. Such single guide RNA (sgRNA) can be synthesized from strong polymerase III promoters (*H1* or *U6*) and efficiently guide Cas9 to their targets [7].

Nowadays, gRNAs can be predicted by different web-based tools [8]. However, not every putative gRNA exhibits the predicted on-target activity, since (i). different cell types may exhibit varying gRNA efficacy [9,10] due to the epigenetic status of the target locus [11]. For example, methylation status can impair Cas9 activity and subsequent DNA repair by altering local chromatin structure [12]. (ii). Furthermore, the cell cycle phase of the target cell could also influence the effectiveness of gRNA [13,14]. (iii). Genomic stability differences in various cell types might also contribute to gRNA effectivity [9,[15], [16], [17], [18]. (iv). Another important consideration about gRNA efficiency is the DNA sequences flanking the PAM. Such context differences might impact both binding and cleavage efficiency of Cas9 due to its “sliding” towards PAMs, thus profoundly impacting gRNA activities [19]. (v). gRNAs designed using computer models may sometimes encounter challenges related to secondary structure and stability, which could potentially result in suboptimal design quality. As such, unstable sgRNAs may fail to effectively bind to Cas proteins [20]. (vi). In this regard, for example, enrichment of guanines and depletion of adenines renders sgRNAs to be more stable [21].

Therefore, before experimental use, especially *in vivo*, it is crucial to assess their cleavage efficiency at the target locus and identify the most efficient ones *in vitro*. Ideally, the efficiency of such targeting should be analysed within the intact genomic context with spared upstream and downstream sequences, in cells of the same epigenetic context (specific both for the stage of ontogenesis as well as tissue/cell specificity). Only these two contextual conditions might guarantee the effective translation to the *in vivo* state. However, it is not always possible—as in case of *in situ* CRISPR-Cas9 techniques [22,23]— or practical to fulfill such strict criteria. Scientists employ various assays to validate predicted gRNAs, including the single-strand annealing (SSA) assay, T7 endonuclease I (T7EI) assay, Sanger sequencing, and western blot [24], [25], [26], [27]. The SSA assay combines luciferase analysis to enable high-throughput evaluation of nuclease activity, while the T7EI assay utilizes the structure-selective enzyme T7 endonuclease I to detect DNA structural deformities, providing a simple and cost-effective method for assessing genome targeting efficiency [28]. Sanger sequencing with inference of CRISPR edits (ICE, [29]) directly examines gRNA target efficiency with precise results, but it is not scalable for screening of on-target efficiencies in multiple gRNAs [26] and cannot be used for *in situ* CRISPR-Cas9 technique [22,23]. Indeed, the latter alters genomes of multiple cells in a defined population producing different indels and other types of edits, but does not produce a single clone for further *in vitro* or *in vivo* application [30]. Moreover, Sanger sequencing is relatively costly and operationally complex [31]. Similarly, western blot, while being a simple and widely used technique, is less precise for determining gRNA targeting efficiency and involves the extraction of whole-cell proteins and analysis of target protein expression levels [27]. All these methods are time-consuming, involve multiple steps and sometimes cannot produce solid results. For example, the SSA technique is very effective, but has been used mostly for TALENs and ZFNs, involves co-transfection with target plasmids and nucleases and requires longer time to perform [24]. GUIDE-seq, similar to Sanger sequencing and SSA assay, takes in consideration the chromosomal context and can assess both on-target and off-target cleavage by introducing specialized DNA adapters or oligonucleotides at the sites of DNA double-strand breaks (DSBs) followed by high-throughput sequencing and analysis of those DNA fragments [32]. However, it also cannot be used for direct on-target efficiency analyses for *in situ* CRISPR-Cas9 approach for the same reason described above [22,23]. Another limitation of GUIDE-seq is that it requires transfection with double-stranded oligodeoxynucleotide (dsODN) tags, not tolerable by some cell types [33]. For example, human hematopoietic stem cells or induced pluripotent stem cells reveal pronounced DNA damage response and undergo apoptosis upon high levels of free DNA ends [34].

A successful CRISPR gene editing application depends greatly on the selection of highly efficient gRNAs. Our aim was to develop an efficient and rapid all-in-one system to check gRNA on-target efficiency in less time with more precision. Here, we describe Noodles, a versatile plasmid, which can benefit researchers and biotechnologists working with CRISPR-Cas9 system.

## Method details

### Principle of the noodles system

**Fig. 1:**
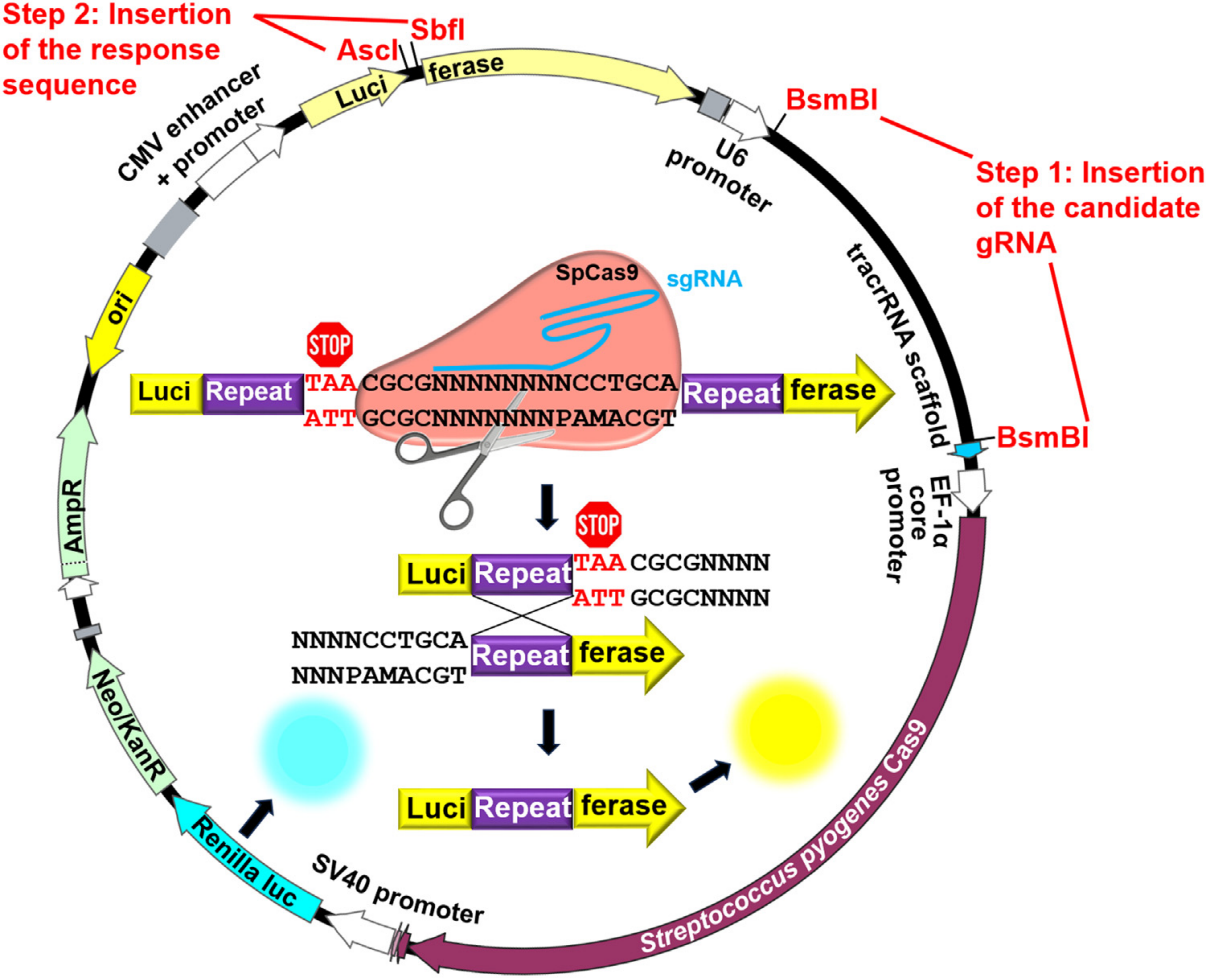
Noodles, the all-in-one system for analysis of CRISPR gRNA on-target efficiency.

To rapidly and sensitively analyze the on-target efficiency of CRISPR gRNAs, we developed the all-in-one plasmid that we designated here as Noodles. In analogy with the predecessor plasmid [1] the system uses advantage of the homology recombination-dependent repair of the split luciferase gene and the resulting luminescence as a readout for CRISPR gRNA-dependent nuclease cleavage of the target region by spCas9 (Fig. 1, [Please insert a reference to our Suppl. data file here]). Since the firefly luciferase is split by the cassette containing <*in-frame repeat***>**<*stop codon***>**<*gRNA response sequence***>**<*in-frame repeat***>**, cleavage of the response sequence by Cas9 restores the luciferase gene via single-strand annealing repair mechanism. In contrast to the analogous plasmids [1], our system comprises the following essential components:

• *SpCas9* gene expression cassette,
• sgRNA expression cassette,
• *Firefly luciferase* gene expression cassette split by the response sequence-containing cassette,
• *Renilla luciferase* gene expression cassette as a transfection efficiency control in the dual-luciferase assay.

### Brief overview of the method

The candidate gRNA pre-selected using the protocol described in section **Design of sgRNAs** (see below) should be subcloned to the Noodles plasmid via sticky end ligation of the BsmBI-opened Noodles with the pre-annealed phosphorylated 〈*g-oligos〉* (sections **Phosphorylation and annealing of the oligonucleotides** and **Subcloning of gRNAs and response sequences to Noodles**, Table 1) to generate the Control plasmid. The latter also serves as a template for subcloning the response sequence for the candidate gRNA to generate the Tester plasmid. Notably, in contrast to the gRNA subcloning step, this response sequence should contain PAM after insertion. In analogy with the previous step, the AscI/SbfI-opened Control plasmid should be ligated with the pre-annealed phosphorylated 〈*rs-oligos〉* (sections **Phosphorylation and annealing of the oligonucleotides** and **Subcloning of gRNAs and response sequences to Noodles**, Table 1). The original 14,197 bp Noodles is thus converted to 12,053 bp Control and Tester plasmids. The subsequent dual luciferase assay will be described in detail as well (see section **Cell transfection and dual luciferase assay**). In addition to the previous use-case examples [22,23], this study also describes an independent validation of the method (see below).

**Table 1:** Template sequences of oligonucleotides for subcloning of gRNA and response sequence.

	<*g-oligos*> for subcloning of gRNA into BsmBI-opened Noodles plasmid to generate the Control plasmid‡	<*rs-oligos*> for subcloning of response sequence into AscI/SbfI -opened Control plasmid to generate the Tester plasmid§
Annealing structure (sense above, antisense below)	5′-^CACC^***G***NNNNNNNNNNNNNNNNNNN-3′ 3′-***C***NNNNNNNNNNNNNNNNNNN^CAAA^-5′	5′-^CGCG^NNNNNNNNNNNNNNNNNNNN**NGG**^TGCA^-3′ 3′-NNNNNNNNNNNNNNNNNNNN**NCC**-5′
5′⇨3′ template sequence for ordering the sense oligonucleotides and sequences for validation	^CACC^***G***NNNNNNNNNNNNNNNNNNN GR1:^CACC^***G***AAGCTTCGGGATGCCATTAT GR2:^CACC^***G***AGGTGGTCCCGTTGCTGTGG GR3:^CACC^***G***TTAAGCTTCCATTACCTTCC GR4:^CACC^***G***CAGCACAATTACCTTTGTGC	^CGCG^NNNNNNNNNNNNNNNNNNNN**NGG**^TGCA^ GR1:^CGCG^AAGCTTCGGGATGCCATTAT**GGG**^TGCA^ GR2:^CGCG^AGGTGGTCCCGTTGCTGTGG**AGG**^TGCA^ GR3:^CGCG^TTAAGCTTCCATTACCTTCC**AGG**^TGCA^ GR4:^CGCG^CAGCACAATTACCTTTGTGC**TGG**^TGCA^
5′⇨3′ template sequence for ordering the antisense oligonucleotides and sequences for validation	^AAAC^NNNNNNNNNNNNNNNNNNNN***C*** GR1:^AAAC^ATAATGGCATCCCGAAGCTT***C*** GR2:^AAAC^CCACAGCAACGGGACCACCT***C*** GR3:^AAAC^GGAAGGTAATGGAAGCTTAA***C*** GR4:^AAAC^GCACAAAGGTAATTGTGCTG***C***	**CCN**NNNNNNNNNNNNNNNNNNNN GR1:**CCC**ATAATGGCATCCCGAAGCTT GR2:**CCT**CCACAGCAACGGGACCACCT GR3:**CCT**GGAAGGTAATGGAAGCTTAA GR4:**CCA**GCACAAAGGTAATTGTGCTG

Overhangs for sticky ends ligation are indicated with superscript letters. For simplicity, we designate here the oligonucleotides containing gRNA or response sequence in direct orientation as sense, while in opposite orientation as antisense.

‡ , IMPORTANT! Add an additional G (indicated by bold italic letter) after the overhang at the start of the sense gRNA oligonucleotide and additional C at the end of the antisense oligonucleotide, but ONLY if gRNA does not already contain the starting G. This ensures that gRNA can be effectively transcribed by RNA Polymerase III.

§ , PAM (only present in the response sequence oligonucleotides) is outlined by bold letters. IMPORTANT! The N in the PAM in 〈*rs-oligos〉* must correspond to the same nucleotide in the PAM of the target gene for the researcher's project. Order the oligonucleotides for subcloning of candidate predicted gRNAs and the corresponding response sequences (use the template format outlined in Table 1) and proceed with the next steps (see below).

### Design of sgRNAs

#### Tools

• ATUM: www.atum.bio
• CHOPCHOP: http://chopchop.cbu.uib.no/
• Crispr-era: http://crispr-era.stanford.edu/

#### Procedure

(1) Predict gRNA using tools such as ATUM, CHOPCHOP, or Crispr-era.
(2) Select the optimal gRNAs based on the best scores provided by the tool, taking into consideration minimization of off-target activities and maximization of the predicted on-target activity, selectivity and specificity.

### Phosphorylation and annealing of the oligonucleotides

#### Materials and reagents

• Sense oligonucleotide (see Table 1).
• Antisense oligonucleotide.
• T4 phage polynucleotide kinase (PNK, NEB, M0201S, see Table S1 for detailed information about reagents).
• T4 phage ligation buffer (NEB: B0202S).
• ddH_2_O (double-distilled ultra-pure and sterile water).

#### Procedure

(1) Reconstitute each sense and antisense oligonucleotide in required amount of ddH_2_O to get a 0.1 mM stock solution.
(2) For each 〈*g-oligos〉* or 〈*rs-oligos〉* (see Table 1), mix the sense and antisense oligonucleotides to set up the phosphorylation reaction mix (Table 2).

**Table 2:** Phosphorylation reaction mixture.

Reagent	Amount per 1 reaction
Sense oligonucleotide [100 µmol]	0.5 µl
Antisense oligonucleotide [100 µmol]	0.5 µl
T4 PNK	0.2 µl
T4 ligation buffer	0.5 µl
ddH_2_O	3.3 µl
(3) Phosphorylate the oligonucleotide in a thermocycler at 37 °C for 30 min.
(4) Perform a denaturation step at 95 °C for 5 min.
(5) Anneal the oligonucleotides by gradually cooling the mixture to 25 °C at a rate of 5 °C per minute.
(6) Dilute annealed oligonucleotides 250-fold by adding 2 µl of the reaction mixture to 498 µl of ddH_2_O.

### Subcloning of gRNAs and response sequences to noodles

#### Materials and reagents

• Noodles plasmid.
• Annealed oligonucleotides from the previous step.
• 10X FastDigest (FD) buffer (see Table S1 for detailed information about reagents).
• 10 mM DTT.
• 10 mM ATP.
• FD BsmBI, FD AscI, FD SbfI.
• T4 DNA Ligase.
• DH5-α cells.
• SOC medium.

#### Procedure

##### Generation of the control plasmid

(1) To subclone gRNA to **Noodles** and thus generate the **Control** plasmid, prepare the following mixture (Table 3):

**Table 3:** Mixture for simultaneous restriction of the Noodles plasmid and ligation with 〈*g-oligos〉* to generate the Control plasmid.

Reagent	Amount per 1 reaction
Noodles (100 ng)	0.3 µl
Annealed 〈*g-oligos〉* (diluted 1:250)	1 µl
10x FD buffer	1 µl
DTT [10 mM]	0.5 µl
ATP [10 mM]	0.5 µl
BsmBI enzyme	0.5 µl
T4 DNA ligase (Roche, K1422)	0.25 µl
ddH_2_O	5.95 µl
(2) Incubate the mixture in a thermocycler at 37 °C for 1 hour.
(3) Transformed 2.5 µl of the reaction mix into 50 µl chemically competent DH5-α cells (Beyotime, D1031M).
(4) Plate the transformed cells on LB agar-ampicillin plates and incubate overnight.

##### Selection and validation of the control plasmid

(1) To verify the successful subcloning of the gRNA, perform colony PCR using the respective sense oligonucleotide for 〈*g-oligos〉* from section **Phosphorylation and annealing of the oligonucleotides** as a forward primer and ATCATGGGAAATAGGCCCTC (Table S2) as the common reverse primer with the following cycler conditions:
  $$\frac{95^{\circ }C}{5m};{\left(\frac{95^{\circ }C}{30s};\frac{55^{\circ }C}{30s};\frac{72^{\circ }C}{35s}\right)}_{25};\frac{8^{\circ }C}{\infty }$$
(2) Prepare the following reaction mixture (Table 4):

**Table 4:** PCR mixture for validation of Control plasmid generation.

Reagent	Amount per 1 reaction
The same sense oligonucleotide for 〈*g-oligos〉* from section Phosphorylation and annealing of the oligonucleotides, (Table 1), 0.1 mM stock	1 µl
Common reverse Primer-R (ATCATGGGAAATAGGCCCTC), 0.1 mM stock	1 µl
Colony or 200 ng **Noodles** plasmid (as a negative control)	1 µl
2x Hieff PCR Master Mix	12.5 µl
ddH_2_O	9.5 µl

Positive band = 363 bp indicates the correctly subcloned gRNA.
(3) Notably, this Colony PCR strategy allows testing multiple clones in one PCR tube following identification of the positive reaction and repeat of the colony PCR with single clones per tube. However, the simultaneous restriction-digestion is highly efficient resulting in majority of clones being positive.
(4) After submerging the toothpick or pipette tip with a colony into the PCR tube with the mix, keep it at 4 °C in a small volume of LB-ampicillin until the PCR results indicate the positive clone that can be grown overnight, validated by Sanger sequencing and used for the next steps.
(5) Control plasmid will be used for the Dual luciferase assay, as well as for subsequent steps to generate the Tester plasmid.

##### Generation of the tester plasmid

(1) To subclone the response sequence (the same sequence targeted by selected gRNA in your gene of interest, including PAM) to Control plasmid and thus generate the Tester plasmid, prepare the following mixture (Table 5):

**Table 5:** Mixture for simultaneous restriction of the Control plasmid and ligation with the 〈*rs-oligos〉* to generate the Tester plasmid.

Reagent	Amount per 1 reaction
Control plasmid (100 ng)	0.3 µl
Annealed 〈 *rs-oligos〉* (diluted 1:250)	1 µl
10x FD buffer	1 µl
DTT [10 mM]	0.5 µl
ATP [10 mM]	0.5 µl
AscI, SbfI enzyme	0.5 µl
T4 DNA ligase (Roche, K1422)	0.25 µl
ddH_2_O	5.95 µl
(2) Incubate the mixture in a thermocycler at 37 °C for 1 hour.
(3) Transformed 2.5 µl of the reaction mix into 50 µl chemically competent DH5-α cells (Beyotime, D1031M).
(4) Plate the transformed cells on LB agar-ampicillin plates and incubate overnight.

##### Selection and validation of the tester plasmid

(1) To verify the successful subcloning of the gRNA, perform colony PCR using the respective sense oligonucleotide for 〈*rs-oligos〉* from section **2.4**. as a forward primer and ACCACGCTGAGGATAGCGGTG (Table S2) as the common reverse primer with the following cycler conditions:
  $$\frac{95^{\circ }C}{5m};{\left(\frac{95^{\circ }C}{30s};\frac{55^{\circ }C}{30s};\frac{72^{\circ }C}{35s}\right)}_{25};\frac{8^{\circ }C}{\infty }$$
(2) Prepare the following reaction mixture (Table 6):

**Table 6:** PCR mixture for validation of Tester plasmid generation.

Reagent	Amount per 1 reaction
Primer-F (the same sense oligonucleotide for 〈*rs-oligos〉* from section 2.4)	1 µl
Primer-R (ACCACGCTGAGGATAGCGGTG)	1 µl
Template (Tester plasmid), 200 ng	1 µl
2 × Hieff PCR Master Mix	12.5 µl
ddH_2_O	9.5 µl

Positive band = 504 bp indicates the correctly subcloned response sequence.
(3) Verify the positive Tester plasmid colony(ies) by Sanger sequencing and proceed to the next step.

### Cell transfection and dual luciferase assay

#### Materials and reagents

• HEK-293T cells (see Table S1 for detailed information about reagents).
• DMEM.
• 10% FBS.
• Opti-MEM medium.
• Opti-MEM Reduced Serum Medium.
• 1X lysis buffer.
• Lipofectamine 2000 Transfection Reagent.
• 96 well plate.

#### Procedure

##### Transfection

**Fig. 2:**
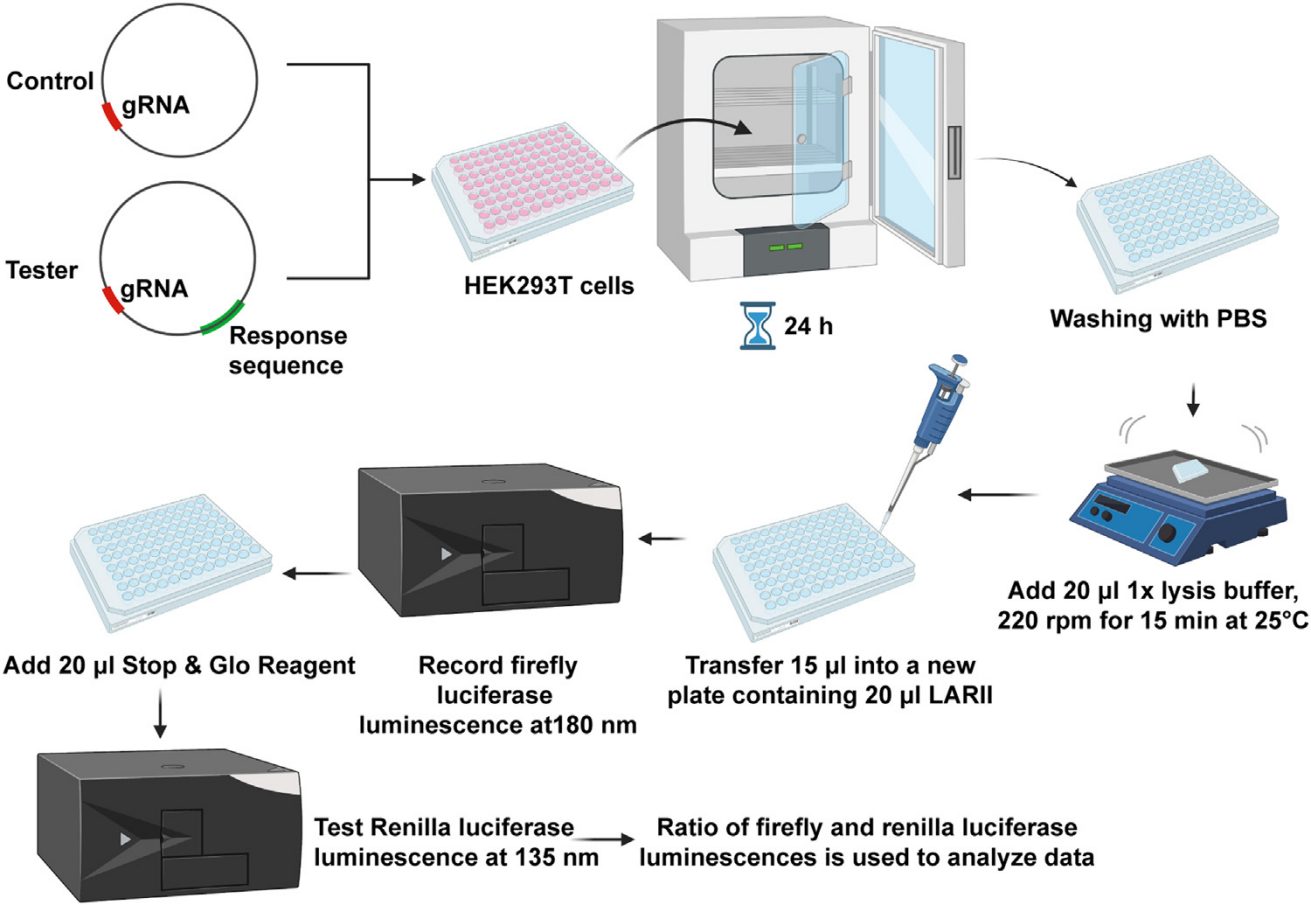
Cell transfection and double luciferase reporter gene assay.
(2) On day 2, at approximately 80%−90% confluence, prepare DNA-lipid complexes incubating the mixture of Solution A (0.1 µg Control or Tester plasmid and 5 µl Opti-MEM medium) B (0.5 µl Lipofectamine and 5 µl of Opti-MEM medium) at 25 °C for 5 min.
(3) Add the DNA-lipid complex to the cells along with 100 µl of fresh DMEM supplemented with 10% FBS.
(4) Incubate the cells for 24 h at 37 °C and 5% CO_2_.

(1) On day 1, plate HEK-293T cells into a 96-well culture plate with DMEM supplemented with 10% FBS at a density of 10,000 cells/well (Fig. 2).

##### Dual luciferase assay

(1) Perform the Dual-Glo Luciferase Assay (Promega, E1960) according to the manufacturer's instructions.
(2) Briefly, add 20 µl/well of 1X lysis buffer to cold PBS-washed cells, incubate for 15 min with shaking at 25 °C.
(3) Transfer 15 µl samples to an opaque 96-well plate, add 20 µl of LARII reagent, mix and measured firefly luminescence at 180 nm.
(4) Next, added 20 µl of Dual-Glo Stop & Glo Reagent mix and measure renilla luminescence at 135 nm.
(5) Calculate the firefly to renilla luminescence ratio, average replicate measurements and analyze the data (Fig. 2).

##### Assay notes

• Aliquot LARII regent to avoid repeated freeze-thaw cycles.
• Prepare Stop & Glo Reagent before each use.
• Dilute cell lysate with the Passive Lysis Buffer if luminescence intensity is exceeding the luminometer measurement range.

## Addendum I. Generation of the noodles plasmid

### Source plasmids

• pmirGLO Dual-Luciferase miRNA Target Expression vector (Promega, E1330);
• 1647_pGL4.51 TLV41 SSA-Luc cloning vector (a generous gift of Gang Bao, Addgene Plasmid #132962, [1]);
• lentiCRISPRv2 puro vector (Addgene Plasmid #98290, [35]).

### Procedure

#### Subcloning of the split luciferase cassette into the renilla luciferase-containing backbone

**Fig. 3:**
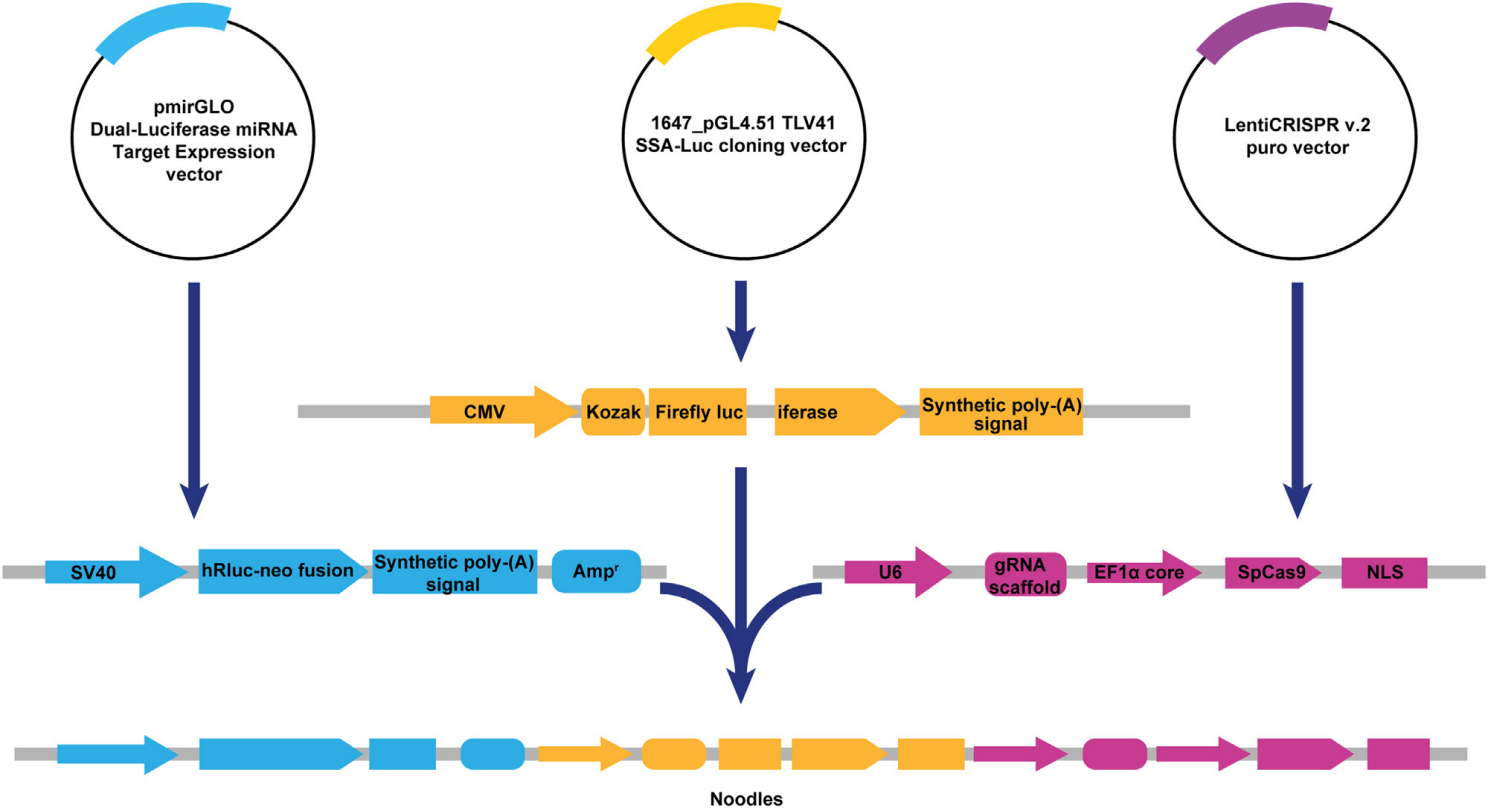
Construction of the Noodles plasmid.
(2) The infusion reaction of the amplified fragment was set up with the gel-extracted BglII/MfeI-opened pmirGLO Dual-Luciferase miRNA Target Expression at 50 °C for 15 min to generate the Intermediate plasmid (Fig. 2).
(3) Then, 2.5 µl of the reaction mix was transformed into 50 µl of chemically competent DH5-α cells (Beyotime, D1031M).
(4) The transformed cells were plated on LB agar-ampicillin plates and incubated overnight at 37 °C.
(5) Next day, selected colonies were transferred to ampicillin-containing LB medium for overnight culture at 37 °C followed by miniprep (Qiagen, 27104), analytical restriction digest with BglII, EcoRV, KpnI, and BamHI.
(6) The positive clones of the Intermediate plasmid were then validated by Sanger sequencing.

(1) Primers to amplify the 〈*split Luc cassette〉* from the 1647_pGL4.51 TLV41 SSA-Luc by PrimeSTAR® Max DNA Polymerase kit (TaKaRa, R045Q) were designed using the Takara infusion cloning primer tool (https://www.takarabio.com/learning-centers/cloning/primer-design-and-other-tools, Table S2, Fig. 3).

#### Subcloning of the cas9-sgrna cassette to generate noodles

(1) Next, PCR-amplified 〈*SpCas9-sgRNA cassette〉* from lentiCRISPRv2 puro was inserted into the MfeI/KpnI-opened Intermediate plasmid by infusion cloning.
(2) Following transformation, overnight culture and analytical restriction digest by EcoRV, HindIII and BglII, the selected positive clone of the resulting Noodles plasmid was verified by Sanger sequencing using the primer walking strategy (Fig. 3).

## Addendum II. Method validation

### Materials and reagents

• HEK-293T cell.
• 3T3L1 cell.
• TRIzol.
• PrimeScript RT reagent Kit.
• Hieff qPCR SYBR Green Master Mix.
• COMPLETE protease inhibitor and phosphatase inhibitor cocktail.
• Loading dye.
• RIPA lysis buffer.
• Polyvinylidene difluoride membrane.
• 5% bovine serum albumin.
• First antibody, second antibody.
• GR antibody.
• β-Actin antibody.

### Procedure

**Fig. 4:**
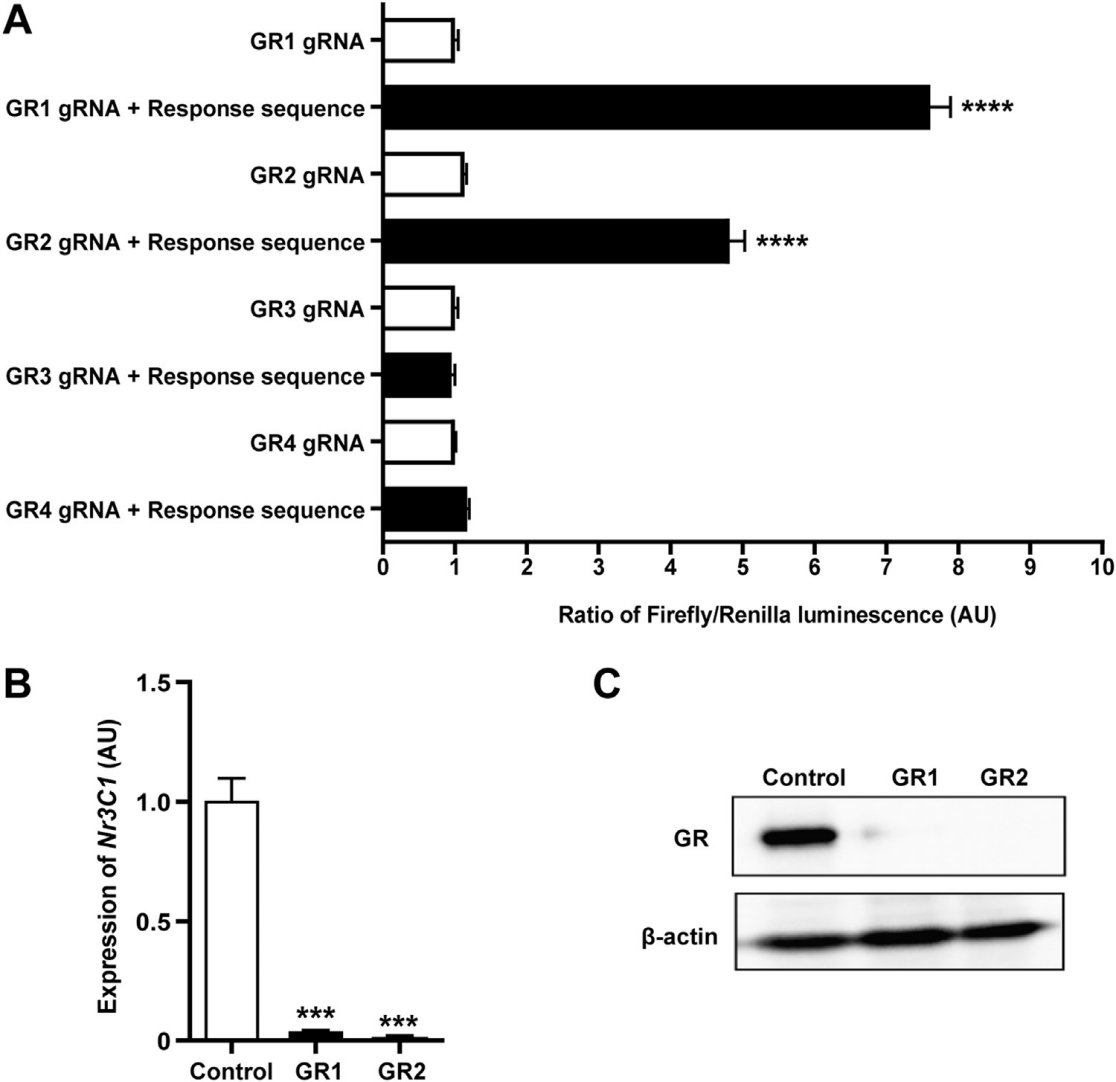
Validation of gRNAs targeting the mouse *glucocorticoid receptor* gene. (A) Dual-luciferase assay-assisted analysis of on-target activity of gRNAs targeting GR using Noodles. *n* = 5 (B, C) Independent *in vitro* verification of the on-target activity of the selected gRNAs by Qpcr, *n* = 3 (B) and western blot (C). All data are expressed as mean ± SEM. ***, *p* < 0.001; ****, *p* < 0.0001 as assessed by unpaired two-tailed Student's *t*-test.
(3) To validate the on-target efficiency independently, we inserted GR1 or GR2 gRNA sequences into HP180 vector (a generous gift from Hui Yang), followed by transfection into 3T3L1 cell line and FACS-sorting after 3 days. The single clone was grown for 3 weeks to generate the stable GR-ko cell line.
(4) Both GR1 and GR2 gRNAs demonstrated reduction of GR expression on mRNA and protein levels (Fig. 4B, C).

(1) To validate our system, we designed multiple gRNAs targeting the mouse glucocorticoid receptor (GR) gene *Nr3C1* as described in section **Design of sgRNAs** (Table 1). A Control and Tester plasmids were generated for each gRNA as described in sections **Phosphorylation and annealing of the oligonucleotides** and **Subcloning of gRNAs and response sequences to Noodles**, followed by transfection into HEK-293T cells and luciferase assay (see section **Cell transfection and dual luciferase assay**).
(2) Two out of the four predicted gRNAs (GR1 and GR2) efficiently guided Cas9-assisted cleavage of the response sequence (Fig. 4A). See **Background** Section for possible reasons why the other two putative gRNAs could be ineffective.

### Methodological notes

(1) All experiments and data shown in this work have been repeated at least twice with consistent results. The presented data were performed on biologically independent samples (*n* = 3). Statistical analyses were performed in GraphPad Prism v.6.0. Unpaired two-tailed Student's *t*-tests were used for comparisons between groups and differences with *p* < 0.05 were considered statistically significant. Data are presented as means  ±  SEM.
(2) Total RNA was extracted by TRIzol (Invitrogen) and quantified using a Nanodrop 2000 ultraviolet (Eppendorf). cDNA was prepared using 1 µg total RNA by reverse transcription PCR using PrimeScript RT reagent Kit (Takara), qPCR was performed using Hieff qPCR SYBR Green Master Mix (Yeasen) with the CFX96 Real-Time PCR Detection System (Bio-Rad). mRNA expression was calculated using the ΔΔCt method with mouse *β-Actin* as the endogenous control. Primer sequences are provided in Table S2.
(3) Whole-cell lysates were extracted using RIPA lysis buffer supplemented with complete protease inhibitor and phosphatase inhibitor cocktail (MCE). Proteins were diluted in loading dye (Yeasen), heated at 95  °C for 10 min and separated on 12% polyacrylamide gel followed by a transfer onto a polyvinylidene difluoride membrane (Millipore). Next, the membranes were blocked with 5% bovine serum albumin for 1 h at 25 °C and incubated with the primary antibodies (dilution 1:1,000 for GR and 1:5,000 for β-Actin, respectively) overnight at 4 °C. Following the incubation with the secondary antibodies (Proteintech) for 1 h at 25 °C, chemiluminescent (Advast) signals were visualised by Biorad ChemiDoc Touch.

## Addendum III. Limitations of the noodles system

Since Noodles requires two cloning steps, it can be classified as a 'medium throughput' method that could test a few dozen gRNAs within a reasonable amount of effort for an independent lab without extensive robotic automation. The major limitation of the method is that it assumes the editing efficiency will be the same in an extra-chromosomal plasmid as it is at the locus within the genome. That locus may be wrapped in chromatin and inaccessible for editing, where a gRNA targeting a different region of the same gene will not be similarly blocked. Noodles does not account for epigenetic factors that may impact Cas9 cutting efficiency. Hence, unless the researchers intend to use *in situ* CRISPR-Cas9 or other techniques not allowing to produce and expand a single clone, the methods like Sanger sequencing, SSA assay or GUIDE-seq represent great alternatives for assessing the gRNA efficiency.

## Ethics statements

No human or animal and no social media platform data were used in this study.

## Related research article

Murgia, N., Y. Ma, S. S. Najam, Y. Liu, J. Przybys, C. Guo, W. Konopka and I. A. Vinnikov (2022). “*In Vivo* Reductionist Approach Identifies miR-15a Protecting Mice From Obesity.” Front Endocrinol 13: 867,929.

## CRediT authorship contribution statement

**Dongfa Lin:** Conceptualization, Formal analysis, Investigation, Project administration, Software, Supervision, Writing – original draft, Writing – review & editing. **Syeda Sadia Najam:** Conceptualization, Formal analysis, Investigation, Project administration, Software, Supervision, Writing – original draft, Writing – review & editing. **Yu Liu:** Conceptualization, Formal analysis, Investigation, Project administration, Software, Supervision, Writing – original draft, Writing – review & editing. **Nicola Murgia:** Conceptualization, Formal analysis, Investigation, Project administration, Software, Supervision, Writing – original draft, Writing – review & editing. **Ilya A. Vinnikov:** Conceptualization, Formal analysis, Investigation, Project administration, Software, Supervision, Writing – original draft, Writing – review & editing.

## Declaration of Competing Interest

The authors declare that they have no known competing financial interests or personal relationships that could have appeared to influence the work reported in this paper.

## Data Availability

No data was used for the research described in the article.

## Appendix Supplementary materials

Full sequence of the Noodles plasmid. Table S1. Cells, enzymes and other reagents used in this study. Table S2. Primers used in this study. Image, application 1
