# Supplementary material for: Noodles, the all-in-one system for on-target efficiency analysis of CRISPR guide RNAs
Source: MethodsX. 2023 Nov 15;12:102481. doi: 10.1016/j.mex.2023.102481 (PMC10757036; doi:10.1016/j.mex.2023.102481)
Supplement: Supplementary file 1 — Full sequence of the Noodles plasmid. Table S1. Cells, enzymes and other reagents used in this study. Table S2. Primers used in this study. [file mmc1.pdf]

Supplementary information for

Article information

Article title

Noodles, the all-in-one system for on-target efficiency analysis of CRISPR guide RNAs

Authors

Dongfa Lin, Syeda Sadia Najam, Yu Liu, Nicola Murgia, Ilya A. Vinnikov*

Corresponding author’s email address and Twitter handle

* Correspondence should be addressed to IAV, i.vinnikov@sjtu.edu.cn, twitter: @vinnikovLAB

Contents:

Full sequence of the Noodles plasmid.

Table S1. Cells, enzymes, and other reagents used in this study.

Table S2. Primers used in this study.

Full sequence of the Noodles plasmid.

cttctgaggcggaaagaaccagctgtggaatgtgtgtcagttagggtgtggaaagtccccaggctccccagcaggcagaa
gtatgcaaagcatgcatctcaattagtcagcaaccaggtgtggaaagtccccaggctccccagcaggcagaagtatgcaa
agcatgcatctcaattagtcagcaaccatagtcccgcccctaactccgcccatcccgcccctaactccgcccagttccgc
ccattctccgccccatggctgactaattttttttatttatgcagaggccgaggccgcctcggcctctgagctattccaga
agtagtgaggaggcttttttggaggcctaggcttttgcaaaaagcttgattcttctgacacaacagtctcgaaccaaagg
ctggagccaccatggcttccaaggtgtacgaccccgagcaacgcaaacgcatgatcactgggcctcagtggtgggctcgc
tgcaagcaaatgaacgtgctggactccttcatcaactactatgattccgagaagcacgccgagaacgccgtgatttttct
gcatggtaacgctgcctccagctacctgtggaggcacgtcgtgcctcacatcgagcccgtggctagatgcatcatccctg
atctgatcggaatgggtaagtccggcaagagcgggaatggctcatatcgcctcctggatcactacaagtacctcaccgct
tggttcgagctgctgaaccttccaaagaaaatcatctttgtgggccacgactgggggcttgtctggcctttcactactc
ctacgagcaccaagacaagatcaaggccatcgtccatgctgagagtgtcgtggacgtgatcgagtcctgggacgagtggc
ctgacatcgaggaggatatcgccctgatcaagagcgaagagggcgagaaaatggtgcttgagaataacttcttcgtcgag
accatgctcccaagcaagatcatgcggaaactggagcctgaggagttcgctgcctacctggagccattcaaggagaaggg
cgaggttagacggcctaccctctcctggcctcgcgagatccctctcgttaagggaggcaagcccgacgtcgtccagattg
tccgcaactacaacgcctaccttcgggccagcgacgatctgcctaagatgttcatcgagtccgaccctgggttcttttcc
aacgctattgtcgagggagctaagaagttccctaacaccgagttcgtgaaggtgaagggcctccacttcagccaggagga
cgctccagatgaaatgggtaagtacatcaagagcttcgtggagcgcgtgctgaagaacgagcagaccggtggtgggagcg
gaggtggcggatcaggtggcggaggctccggagggattgaacaagatggattgcacgcaggttctccggccgcttgggtg
gagaggctattcggctatgactgggcacaacagacaatcggctgctctgatgccgccgtgttccggctgtcagcgcaggg
gcgcccggttctttttgtcaagaccgacctgtccggtgccctgaatgaactgcaggacgaggcagcgcggctatcgtggc
tggccacgacgggcgttccttgcgcagctgtgctcgacgttgtcactgaagcgggaagggactggctgctattgggcgaa
gtgccggggcaggatctcctgtcatctcaccttgctcctgccgagaaagtatccatcatggctgatgcaatgcggcggct
gcatacgcttgatccggctacctgcccattcgaccaccaagcgaaacatcgcatcgagcgagcacgtactcggatggaag
ccggtcttgtcgatcaggatgatctggacgaagagcatcaggggctcgcgccagccgaactgttcgccaggctcaaggcg
cgcatgcccgacggcgaggatctcgtcgtgacccatggcgatgcctgcttgccgaatatcatggtggaaaatggccgctt
ttctggattcatcgactgtggccggctgggtgtggcggaccgctatcaggacatagcgttggctacccgtgatattgctg
aagagcttggcggcgaatgggctgaccgcttcctcgtgctttacggtatcgccgctcccgattcgcagcgcatcgccttc
tatcgccttcttgacgagttcttctgagcgggactctggggttcgaaatgaccgaccaagcgacgcccaacctgccatca
cgatggccgcaataaaatatctttattttcattacatctgtgtgttggttttttgtgtgaatcgatagcgataaggatcc

tctttgcgcttgcgttttcccttgtccagatagcccagtagctgacattcatccggggtcagcaccgtttctgcggactg
gctttctacgtaatggtttcttagacgtcaggtggcacttttcggggaaatgtgcgcggaacccctatttgtttattttt
ctaaatacattcaaatatgtatccgctcatgagacaataaccctgataaatgcttcaataatattgaaaaaggaagagta
tgagtattcaacatttccgtgtcgcccttattcccttttttgcggcattttgccttcctgtttttgctcacccagaaacg
ctggtgaaagtaaaagatgctgaagatcagttgggtgcacgagtgggttacatcgaactggatctcaacagcggtaagat
ccttgagagttttcgccccgaagaacgttttccaatgatgagcacttttaaagttctgctatgtggcgcggtattatccc
gtattgacgccgggcaagagcaactcggtcgccgcatacactattctcagaatgacttggttgagtactcaccagtcaca
gaaaagcatcttacggatggcatgacagtaagagaattatgcagtgctgccataaccatgagtgataacactgcggccaa
cttacttctgacaactatcggaggaccgaaggagctaaccgcttttttgcacaacatgggggatcatgtaactcgccttg
atcgttgggaaccggagctgaatgaagccataccaaacgacgagcgtgacaccacgatgcctgtagcaatggcaacaacg
ttgcgcaaactattaactggcgaactacttactctagcttcccggcaacaattaatagactggatggaggcggataaagt
tgcaggaccacttctgcgctcggcccttccggctggctggtttattgctgataaatctggagccggtgagcgtgggtctc
gcggtatcattgcagcactggggccagatggtaagccctcccgtatcgtagttatctacacgacggggagtcaggcaact
atggatgaacgaaatagacagatcgctgagataggtgcctcactgattaagcattggtaattcgaaatgaccgaccaagc
gacgcccaaccggtatcagctcactcaaaggcggtaatacggttatccacagaatcaggggataacgcaggaaagaacat
gtgagcaaaaggccagcaaaaggccaggaaccgtaaaaaggccgcgttgctggcgtttttccataggctccgcccccctg
acgagcatcacaaaaatcgacgctcaagtcagaggtggcgaaacccgacaggactataaagataccaggcgtttccccct
ggaagctccctcgtgcgctctcctgttccgaccctgccgcttaccggatacctgtccgcctttctcccttcgggaagcgt
ggcgctttctcatagctcacgctgtaggtatctcagttcggtgtaggtcgttcgctccaagctgggctgtgtgcacgaac
cccccgttcagcccgaccgctgcgccttatccggtaactatcgtcttgagtccaacccggtaagacacgacttatcgcca
ctggcagcagccactggtaacaggattagcagagcgaggtatgtaggcggtgctacagagttcttgaagtggtggcctaa
ctacggctacactagaaggacagtatttggtatctgcgctctgctgaagccagttaccttcggaaaaagagttggtagct
cttgatccggcaaacaaaccaccgctggtagcggtggtttttttgtttgcaagcagcagattacgcgcagaaaaaaagga
tttcaagaagatcctttgatcttttctacggggtctgacgctcagtggaacgaaaactcacgttaagggattttggtcat
gagattatcaaaaaggatcttcacctagatccttttatagtccggaaatacaggaacgcacgctggatggcccttcgctg
ggatggtgaaaccatgaaaaatggcagcttcagtggattaagtgggggtaatgtggcctgtaccctctggttgcataggt
attcatacggttaaaatttatcaggcgcgattgcggcagtttttcgggtggtttgttgccatttttacctgtctgctgcc
gtgatcgcgctgaacgcgttttagcggtgcgtacaattaagggattatggtaaatccacttactgtctgccctcgtagcc
atcgagataaaccgcagtactccggccacgatgcgtccggcgtagaggatcgagatcctagttattaatagtaatcaatt
acggggtcattagttcatagcccatatatggagttccgcgttacataacttacggtaaatggcccgcctggctgaccgcc

caacgacccccgcccattgacgtcaataatgacgtatgttcccatagtaacgccaatagggactttccattgacgtcaat
gggtggagtatttacggtaaactgcccacttggcagtacatcaagtgtatcatatgccaagtccgccccctattgacgtc
aatgacggtaaatggcccgcctggcattatgcccagtacatgaccttacgggactttcctacttggcagtacatctacgt
attagtcatcgctattaccatggtgatgcggttttggcagtacaccaatgggcgtggatagcggtttgactcacggggat
ttccaagtctccaccccattgacgtcaatgggagtttgttttggcaccaaaatcaacgggactttccaaaatgtcgtaat
aaccccgccccgttgacgcaaatgggcggtaggcgtgtacggtgggaggtctatataagcagagctcgtttagtgaaccg
tcagatcactagaagctttattgcggtagtttatcacagttaaattgctaacgcagtcagtgggcctcggcggccaagct
tggcaatccggtactgttggtaaagccaccatggaagatgccaaaaacattaagaagggcccagcgccattctacccact
cgaagacgggaccgccggcgagcagctgcacaaagccatgaagcgctacgccctggtgcccggcaccatcgcctttaccg
acgcacatatcgaggtggacattacctacgccgagtacttcgagatgagcgttcggctggcagaagctatgaagcgctat
gggctgaatacaaaccatcggatcgtggtgtgcagcgagaatagcttgcagttcttcatgcccgtgttgggtgccctgtt
catcggtgtggctgtggccccagctaacgacatctacaacgagcgcgagctgctgaacagcatgggcatcagccagccca
ccgtcgtattcgtgagcaagaaagggctgcaaaagatcctcaacgtgcaaaagaagctaccgatcatacaaaagatcatc
atcatggatagcaagaccgactaccagggcttccaaagcatgtacaccttcgtgacttcccatttgccacccggcttcaa
cgagtacgacttcgtgcccgaataatgaggcgcgaccatcttcttcaaggacgacggcgcgcctgggatcctgcaggtgt
gcagcgagaatagcttgcagttcttcatgcccgtgttgggtgccctgttcatcggtgtggctgtggccccagctaacgac
atctacaacgagcgcgagctgctgaacagcatgggcatcagccagcccaccgtcgtattcgtgagcaagaaagggctgca
aaagatcctcaacgtgcaaaagaagctaccgatcatacaaaagatcatcatcatggatagcaagaccgactaccagggct
tccaaagcatgtacaccttcgtgacttcccatttgccacccggcttcaacgagtacgacttcgtgcccgagagcttcgac
cgggacaaaaccatcgccctgatcatgaacagtagtggcagtaccggattgcccaagggcgtagccctaccgcaccgcac
cgcttgtgtccgattcagtcatgcccgcgaccccatcttcggcaaccagatcatccccgacaccgctatcctcagcgtgg
tgccatttcaccacggcttcggcatgttcaccacgctgggctacttgatctgcggctttcgggtcgtgctcatgtaccgc
ttcgaggaggagctattcttgcgcagcttgcaagactataagattcaatctgccctgctggtgcccacactatttagctt
cttcgctaagagcactctcatcgacaagtacgacctaagcaacttgcacgagatcgccagcggcggggcgccgctcagca
aggaggtaggtgaggccgtggccaaacgcttccacctaccaggcatccgccagggctacggcctgacagaaacaaccagc
gccattctgatcacccccgaaggggacgacaagcctggcgcagtaggcaaggtggtgcccttcttcgaggctaaggtggt
ggacttggacaccggtaagacactgggtgtgaaccagcgcggcgagctgtgcgtccgtggccccatgatcatgagcggct
acgttaacaaccccgaggctacaaacgctctcatcgacaaggacggctggctgcacagcggcgacatcgcctactgggac
gaggacgagcacttcttcatcgtggaccggctgaagagcctgatcaaatacaagggctaccaggtagccccagccgaact
ggagagcatcctgctgcaacaccccaacatcttcgacgccggggtcgccggcctgcccgacgacgatgccggcgagctgc

ccgccgcagtcgtcgtgctggaacacggtaaaaccatgaccgagaaggagatcgtggactatgtggccagccaggttaca
accgccaagaagctgcgcggtggtgttgtgttcgtggacgaggtgcctaaaggactgaccggcaagttggacgcccgcaa
gatccgcgagattctcattaaggccaagaagggcggcaagatcgccgtgtaataattctagagtcggggcggccggccgc
ttcgagcagacatgataagatacattgatgagtttggacaaaccacaactagaatgcagtgaaaaaaatgctttatttgt
gaaatttgtgatgctattgctttatttgtaaccattataagctgcaataaacaagttaacaacaacaattgcgagggcct
atttcccatgattccttcatatttgcatatacgatacaaggctgttagagagataattagaattaatttgactgtaaaca
caaagatattagtacaaaatacgtgacgtagaaagtaataatttcttgggtagtttgcagttttaaaattatgttttaaa
atggactatcatatgcttaccgtaacttgaaagtatttcgatttcttggctttatatatcttgtggaaaggacgaaacac
cggagacggttgtaaatgagcacacaaaatacacatgctaaaatattatattctatgacctttataaaatcaaccaaaat
cttctttttaataactttagtatcaataattagaatttttatgttcctttttgcaaacttttaataaaaatgagcaaaat
aaaaaaacgctagttttagtaactcgcgttgttttcttcacctttaataatagctactccaccacttgttcctaagcggt
cagctcctgcttcaatcatttttgagcatcttcaaatgttctaactccaccagctgctttaactaaagcattgtcttta
acaactgacttcattagtttaacatcttcaaatgttgcacctgattttgaaaatcctgttgatgttttaacaaattctaa
tccagcttcaacagctatttcacaagctttcatgatttcttcttttgttaataaacaattttccataatacatttaacaa
catgtgatccagctgctttttttacagctttcatgtcttctaaaactaattcataatttttgtcttttaatgcaccaata
tttaataccatatcaatttctgttgcaccatctttaattgcttcagaaacttcgaatgcttttgtagctgttgtgcatgc
acctagaggaaaacctacaacatttgttattcctacatttgtgccttttaataattctttacaatagcttgttcaatatg
aattaacacaaactgttgcaaaatcaaattcaattgcttcatcacataattgtttaatttcagctttcgtagcatcttgt
tttaataatgtgtgatctatatatttgtttagtttcatttttctcctatatattcatttttaattttaattctttaata
atttcgtctactttaactttagcgttttgaacagattcaccaacacctataaaataaatttttagtttaggttcagttcc
acttgggcgaacagcaaatcatgacttatcttctaaataaaattttagtaagtcttgtcctggcatattatacattccat
cgatgtagtcttcaacattaacaactttaagtccagcaatttgagttaagggtgttgctctcaatgatttcattaatggt
tcaatttttaatttcttttcttctggtttaaaattcaagtttaaagtgaaagtgtaatatgcacccatttctttaaataa
atcttctaaatagtctactaatgttttattttgtttttataaaatcaagcagcctctgctattaatatagaagcttgta
ttccatctttatctctagctgagtcatcaattacatatccataactttcttcataagcaaaaacaaaatttaatccgtta
tcttcttctttagcaatttctctacccattcatttaaatccagttaaagtttttacaatattaactccatatttttcatg
agcgattctatcacccaaatcacttgttacaaaacttgaatatagagccggatttttggaatgctatttaagcgtttta
gatttgataattttcaatcaattaaaattggtcctgtttgatttccatctaatcttacaaaatgaccatcatgttttatt
gccattccaaatctgtcagcatctgggtcattcataataataatatctgcatcatgtttaataccatattcaagcggtat
ttttcatgcaggatcaaattctggatttggatttacaacatttttaaatgtttcatcttcaaatgcatgctcttcaacct

caataacgttatatcctgattcacgtaatattttggggtaaatttagttcctgttccattaactgcgctaaaaataatt
tttaaatctttttagcttcttgctctttttgtacgtctctgttttagagctagaaatagcaagttaaaataaggctag
tccgttatcaacttgaaaaagtggcaccgagtcggtgctttttgaattcgctagctaggtcttgaaaggagtgggaatt
ggctccggtgcccgtcagtgggcagagcgcacatcgcccacagtccccgagaagttggggggaggggtcggcaattgatc
cggtgcctagagaaggtggcgcggggtaaactgggaaagtgatgtcgtgtactggctccgcctttttcccgagggtgggg
gagaaccgtatataagtgcagtagtcgccgtgaacgttctttttcgcaacgggtttgccgccagaacacaggaccggttc
tagagcgctgccaccatggacaagaagtacagcatcggcctggacatcggcaccaactctgtgggctgggccgtgatcac
cgacgagtacaaggtgcccagcaagaaattcaaggtgctgggcaacaccgaccggcacagcatcaagaagaacctgatcg
gagccctgctgttcgacagcggcgaaacagccgaggccacccggctgaagagaaccgccagaagaagatacaccagacgg
aagaaccggatctgctatctgcaagagatcttcagcaacgagatggccaaggtggacgacagcttcttccacagactgga
agagtccttcctggtggaagaggataagaagcacgagcggcaccccatcttcggcaacatcgtggacgaggtggcctacc
acgagaagtaccccaccatctaccacctgagaaagaaactggtggacagcaccgacaaggccgacctgcggctgatctat
ctggccctggcccacatgatcaagttccggggccacttcctgatcgagggcgacctgaaccccgacaacagcgacgtgga
caagctgttcatccagctggtgcagacctacaaccagctgttcgaggaaaaccccatcaacgccagcggcgtggacgcca
aggccatcctgtctgccagactgagcaagagcagacggctggaaaatctgatcgcccagctgcccggcgagaagaagaat
ggcctgttcggaaacctgattgccctgagcctgggcctgacccccaacttcaagagcaacttcgacctggccgaggatgc
caaactgcagctgagcaaggacacctacgacgacgacctggacaacctgctggcccagatcggcgaccagtacgccgacc
tgtttctggccgccaagaacctgtccgacgccatcctgctgagcgacatcctgagagtgaacaccgagatcaccaaggcc
cccctgagcgcctctatgatcaagagatacgacgagcaccaccaggacctgaccctgctgaaagctctcgtgcggcagca
gctgcctgagaagtacaaagagattttcttcgaccagagcaagaacggctacgccggctacattgacggcggagccagcc
aggaagagttctacaagttcatcaagcccatcctggaaaagatggacggcaccgaggaactgctcgtgaagctgaacaga
gaggacctgctgcggaagcagcggaccttcgacaacggcagcatcccccaccagatccacctgggagagctgcacgccat
tctgcggcggcaggaagatttttacccattcctgaaggacaaccgggaaaagatcgagaagatcctgaccttccgcatcc
cctactacgtgggccctctggccaggggaaacagcagattcgcctggatgaccagaaagagcgaggaaaccatcaccccc
tggaacttcgaggaagtggtggacaagggcgcttccgcccagagcttcatcgagcggatgaccaacttcgataagaacct
gcccaacgagaaggtgctgcccaagcacagcctgctgtacgagtacttcaccgtgtataacgagctgaccaaagtgaaat
acgtgaccgagggaatgagaaagcccgccttcctgagcggcgagcagaaaaaggccatcgtggacctgctgttcaagacc
aaccggaaagtgaccgtgaagcagctgaaagaggactacttcaagaaaatcgagtgcttcgactccgtggaaatctccgg
cgtggaagatcggttcaacgcctccctgggcacataccacgatctgctgaaaattatcaaggacaaggacttcctggaca
atgaggaaaacgaggacattctggaagatatcgtgctgaccctgacactgtttgaggacagagagatgatcgaggaacgg

ctgaaaacctatgcccacctgttcgacgacaaagtgatgaagcagctgaagcggcggagatacaccggctggggcaggct
gagccggaagctgatcaacggcatccgggacaagcagtccggcaagacaatcctggatttcctgaagtccgacggcttcg
ccaacagaaacttcatgcagctgatccacgacgacagcctgacctttaaagaggacatccagaaagcccaggtgtccggc
cagggcgatagcctgcacgagcacattgccaatctggccggcagccccgccattaagaagggcatcctgcagacagtgaa
ggtggtggacgagctcgtgaaagtgatgggccggcacaagcccgagaacatcgtgatcgaaatggccagagagaaccaga
ccacccagaagggacagaagaacagccgcgagagaatgaagcggatcgaagagggcatcaaagagctgggcagccagatc
ctgaaagaacaccccgtggaaaacacccagctgcagaacgagaagctgtacctgtactacctgcagaatgggcgggatat
gtacgtggaccaggaactggacatcaaccggctgtccgactacgatgtggaccatatcgtgcctcagagctttctgaagg
acgactccatcgacaacaaggtgctgaccagaagcgacaagaaccggggcaagagcgacaacgtgccctccgaagaggtc
gtgaagaagatgaagaactactggcggcagctgctgaacgccaagctgattacccagagaaagttcgacaatctgaccaa
ggccgagagaggcggcctgagcgaactggataaggccggcttcatcaagagacagctggtggaaacccggcagatcacaa
agcacgtggcacagatcctggactcccggatgaacactaagtacgacgagaatgacaagctgatccgggaagtgaaagtg
atcaccctgaagtccaagctggtgtccgatttccggaaggatttccagttttacaaagtgcgcgagatcaacaactacca
ccacgcccacgacgcctacctgaacgccgtcgtgggaaccgccctgatcaaaaagtaccctaagctggaaagcgagttcg
tgtacggcgactacaaggtgtacgacgtgcggaagatgatcgccaagagcgagcaggaaatcggcaaggctaccgccaag
tacttcttctacagcaacatcatgaactttttcaagaccgagattaccctggccaacggcgagatccggaagcggcctct
gatcgagacaaacggcgaaaccggggagatcgtgtgggataagggccgggattttgccaccgtgcggaaagtgctgagca
tgccccaagtgaatatcgtgaaaaagaccgaggtgcagacaggcggcttcagcaaagagtctatcctgcccaagaggaac
agcgataagctgatcgccagaaagaaggactgggaccctaagaagtacggcggcttcgacagccccaccgtggcctattc
tgtgctggtggtggccaaagtggaaaagggcaagtccaagaaactgaagagtgtgaaagagctgctggggatcaccatca
tggaaagaagcagcttcgagaagaatcccatcgactttctggaagccaagggctacaaagaagtgaaaaaggacctgatc
atcaagctgcctaagtactccctgttcgagctggaaaacggccggaagagaatgctggcctctgccggcgaactgcagaa
gggaaacgaactggccctgccctccaaatatgtgaacttcctgtacctggccagccactatgagaagctgaagggctccc
ccgaggataatgagcagaaacagctgtttgtggaacagcacaagcactacctggacgagatcatcgagcagatcagcgag
ttctccaagagagtgatcctggccgacgctaatctggacaaagtgctgtccgcctacaacaagcaccgggataagcccat
cagagagcaggccgagaatatcatccacctgtttaccctgaccaatctgggagcccctgccgccttcaagtactttgaca
ccaccatcgaccggaagaggtacaccagcaccaaagaggtgctggacgccaccctgatccaccagagcatcaccggcctg
tacgagacacggatcgacctgtctcagctgggaggcgacaagcgacctgccgccacaaagaaggctggacaggctaagaa
gaagaaagattacaaagacgatgacgataaggggtac

Table S1. Cells, enzymes, and other reagents used in this study.

**Enzyme**	**Company**	**Product Article Number**
SgsI (AscI) (10 U/μL)	Thermo Scientific™	ER1891
SdaI (SbfI) (10 U/μL)	Thermo Scientific™	ER1191
Esp3I (BsmBI) (10 U/μL)	Thermo Scientific™	ER0452
FastDigest BglII	Thermo Scientific™	FD0083
MunI (MfeI) (10 U/μL)	Thermo Scientific™	ER07521
FastDigest KpnI	Thermo Scientific™	FD0524
Eco32I (EcoRV) (10 U/μL)	Thermo Scientific™	ER0301
FastDigest HindIII	Thermo Scientific™	FD0504
FastDigest BamHI	Thermo Scientific™	FD0054
T4 DNA Ligase	TaKaRa	2011B
FastDigest Buffer (10X)	Thermo Scientific™	B64
T4_PNK	NEB	M0201S
T4 ligation buffer	NEB	B0202S
10X FD buffer	ThermoFisher	B64
10 mM DTT	ThermoFisher	15596026
10 mM ATP	ThermoFisher	AM8110G
DH5-α cells	Beytime	D1031S
SOC medium	Sigma-Aldrich	S1797
HEK-293T cells	ATCC	CRL-1573^{TM}
DMEM	ThermoFisher	11965092
10% FBS	ThermoFisher	12483020
Opti-MEM Reduced Serum Medium	ThermoFisher	31985070
1X lysis buffer	ThermoFishe	89901
Lipofectamine 2000 Transfection Reagent	ThermoFisher	11668019
96 well plate	ThermoFisher	442404
3T3L1 cell	ATCC	CL-173^{TM}
TRIzol	ThermoFisher	15596026
PrimeScript RT reagent Kit	Takara	RR047A
Hieff qPCR SYBR Green Master Mix	Yeasen	11184ES08
Complete protease inhibitor and Phosphatase Inhibitor Cocktail	ThermoFishe	78440
RIPA lysis buffer	ThermoFisher	89900
polyvinylidene difluoride membrane	ThermoFisher	88585
5% bovine serum albumin	ThermoFisher	23210
GR antibody (Rabbit mAb)	CST	12041
β -Actin antibody (Mouse / IgM)	Proteintech	60008-1-Ig
HRP-conjugated Affinipure Goat Anti-Mouse IgG(H+L)	Proteintech	SA00001-1

Table S2. Primers used in this study.

ID	Forward (5'⇨3')	Reverse (5'⇨3')	Application
Luc	TAGAGGATCGAGATCCTAGTTATTAATAGTAATCAATTAC	AAAATGAATGCAATTGTTGTTGTTAACTTGTTTATTG	Amplification of the split luciferase cassette from pGL4.51 TLV41 plasmid for infusion cloning
MfeI	TTAACAACAACAATTGAATTCGCTAGCTAGGTCTTGAAAGG	AAAATGAATGCAATTGCCTTATCGTCATCGTCTTTGTAATC	Amplification of sgRNA + Cas9 cassette from lentiCRISPRv2 puro vector plasmid for infusion cloning protocol
MfeI-KpnI	TTAACAACAACAATTGCGAGGGCCTATTTCCCATG	TTTCCGCCTCAGAAGGTACCCCTTATCGTCATCGTCTTTGTAATC	Verification of Noodles plasmid integrity
Gapdh	AGGTCGGTGTGAACGGATTTG	TGTAGACCATGTAGTTGAGGTCA	qPCR primer for *Gapdh*
Nr3c1	AGCTCCCCCTGGTAGAGAC	GGTGAAGACGCAGAAACCTTG	qPCR primer for *Nr3c1*
Control	GR1: CACC**G**AAGCTTCGGGATGCCATTAT<br>GR2: CACC**G**AGGTGGTCCCGTTGCTGTGG<br>GR3: CACC**G**TTAAGCTTCCATTACCTTCC<br>GR4: CACC**G**CAGCACAATTACCTTTGTGC	Common:<br>ATCATGGGAAATAGGCCCTC	Colony PCR screening primer for checking the correctness of the **Control** plasmid.

Tester	GR1: CGCGAAGCTTCGGGATGCCATTAT**GGG**TGCA<br>GR2: CGCGAGGTGGTCCCGTTGCTGTGG**AGG**TGCA<br>GR3: CGCGTTAAGCTTCCATTACCTTCC**AGG**TGCA<br>GR4: CGCGCAGCACAATTACCTTTGTGC**TGG**TGCA	Common:<br>ACCACGCTGAGGATAGCGGTG	Colony PCR screening primer for checking the correctness of the **Tester** plasmid.

Notably, sense oligonucleotides from **Table 1** are used as forward primers in analytical colony PCR. Additionally added G at the 5'-end of the gRNA and PAM sequences are outlined in bold (see **Table 1** for more details).
